# The action of mimetic peptides on connexins protects fibroblasts from the negative effects of ischemia reperfusion

**DOI:** 10.1242/bio.013573

**Published:** 2015-10-15

**Authors:** Beverley J. Glass, Rebecca G. Hu, Anthony R. J. Phillips, David L. Becker

**Affiliations:** 1 Department of Cell and Developmental Biology, University College London, Gower Street, London WC1E 6BT, UK; 2 Lee Kong Chian School of Medicine, Nanyang Technological University, 11 Mandalay Road, Singapore308232; 3 CoDa Therapeutics, Inc., 10 College Hill Road, Herne Bay, Auckland 1011, New Zealand; 4 School of Biological Sciences, University of Auckland, Auckland 1010, New Zealand

**Keywords:** Hemichannel, Gap junction, Ischemia Reperfusion, Mimetic peptide

## Abstract

Connexins have been proposed as a target for therapeutic treatment of a variety of conditions. The main approaches have been by antisense or small peptides specific against connexins. Some of these peptides enhance communication while others interfere with connexin binding partners or bind to the intracellular and extracellular loops of connexins. Here, we explored the mechanism of action of a connexin mimetic peptide by evaluating its effect on gap junction channels, connexin protein levels and hemichannel activity in fibroblast cells under normal conditions and following ischemia reperfusion injury which elevates Cx43 levels, increases hemichannel activity and causes cell death. Our results showed that the effects of the mimetic peptide were concentration-dependent. High concentrations (100-300 μM) significantly reduced Cx43 protein levels and GJIC within 2 h, while these effects did not appear until 6 h when using lower concentrations (10-30 μM). Cell death can be reduced when hemichannel opening and GJIC were minimised.

## Abstract (precis)

**Summary:** Connexin mimetic peptides can reduce the levels of connexin proteins in cells and can prevent the spread of cell death that occurs following ischemia reperfusion injury, which has therapeutic potential.

## INTRODUCTION

Connexin (Cx) modulation has been recognised as a target for therapeutic intervention in a number of diseases (Alldredge, 2008; Bennett, 1994; Ormonde et al., 2012; Wei et al., 2004) and there are now a series of clinical trials targeting Cxs. The principal approaches to functional modulation of Cx have so far been the use of antisense oligodeoxynucleotides (Law et al., 2006; Mori et al., 2006; O'Carroll et al., 2013a,b; Ormonde et al., 2012; Qiu et al., 2003) or small peptides (Chaytor et al., 1997; Evans and Boitano, 2001; Herve and Dhein, 2010; Hunter et al., 2005; Kjolbye et al., 2007; O'Carroll et al., 2008; O'Quinn et al., 2011; Skyschally et al., 2013). Peptide approaches have varied. For example, ACT-1 is a mimetic peptide that reportedly interrupts binding partner interaction with the Cx43 PDZ2 domain (Ghatnekar et al., 2014, 2009; Ongstad et al., 2013). Danegaptide (formerly known as GAP-134) is a dipeptide said to activate gap junction (GJ) communication channels between cells (Skyschally et al., 2013). Mimetic peptides that mimic the extracellular loops of Cxs have also been reported to influence gap junction and hemichannel function (Warner et al., 1995). However, the exact mechanism of action leading to the protective effect seen with Cx mimetic peptides is still undefined (Chen et al., 2015b). Several *in vitro* studies in a broad range of cells and tissues have resulted in three key theories as to how Cx mimetic peptides can interrupt or inhibit GJ intercellular communication (GJIC) (Evans and Boitano, 2001; Evans et al., 2012). These include: (1) Cx mimetic peptide interaction with an undocked hemichannel (CxHC) in the plasma membrane, thereby preventing connexons docking and GJ formation with other cells; (2) interacting with CxHCs or GJs and altering channel gating; (3) interacting via the intercellular space between the GJs leading to dissociation of the GJ plaques and subsequent internalization and breakdown.

Here we explored the mechanism underlying the actions of a mimetic peptide on GJ channels, Cx protein levels, and CxHC activity in fibroblast cells under normal conditions and following ischemia-reperfusion injury. Tissue ischemia is a major medical problem that may occur in a number of organs such as the heart (e.g. cardiac infarction), brain (e.g. ischemic stroke), and skin (e.g. pressure ulcer). The common feature is a period of blood flow restriction to the tissue resulting in deprivation of oxygen, glucose and other nutrients needed for cell survival. The profound damage, however, occurs during the reperfusion phase. This is when the blood supply returns and causes inflammation and oxidative damage to the tissue that has been deprived of oxygen for a period of time (García-Dorado et al., 2004). Often this damage spreads beyond the initial ischemic region and causes cell death in the adjacent area. The spread of cell death has been attributed to GJIC in stroke models (Cotrina et al., 1998) and models of heart attack (García-Dorado et al., 2004) whilst negative effects of CxHC activity on cell viability have been reported in models of stroke (Cotrina et al., 1998; García-Dorado et al., 2004; Orellana et al., 2010; Thompson et al., 2006). The ‘bystander’ effect model suggests that death signals can spread laterally through GJs from dying cells into their healthy neighbour cells (Mao et al., 2009; Zhang et al., 2013). However, some reports also attribute cell death in ischemia-reperfusion models to the opening of undocked CxHC, causing blood vessel leakiness and release of ATP leading to activation of purinergic receptors (Danesh-Meyer et al., 2012; Clarke et al., 2009; Davidson et al., 2013; Orellana et al., 2010; Poornima et al., 2012; Thompson et al., 2006).

Cx mimetic peptides have demonstrated therapeutic benefit for protecting neuronal cells in the event of ischemia reperfusion (Davidson et al., 2013). Application of Cx mimetic peptides can significantly reduce the cell damage that occurs in an *in vitro* and an *in vivo* spinal cord injury model (O'Carroll et al., 2008, 2013a,b). Building on this research, using a model of cerebral ischemia in foetal sheep, Davidson and colleagues demonstrated that Cx mimetic peptide could increase the survival rate of cells during ischemia reperfusion and reduce seizure activity (Davidson et al., 2012). Cardiac protection has also been noted in rat models of myocardial infarction, where Cx mimetic peptides leading to a significant reduction of infarct size by over 60% (Hawat et al., 2012). However, the precise mechanism of action of the peptides is still unknown.

There is no published work of which we are aware indicating that Cx mimetic peptides reduce the extensive progressive damage often seen in pressure ulcers. Repeated cycle of pressure and relief causes severe tissue ischemia reperfusion damage in the skin, similar to the damage seen in cerebral and cardiac ischemia reperfusion. If left untreated, this will ultimately lead to the formation of pressure ulcer and an open wound. There are currently no effective treatments for this irreversible pressure ulceration and understanding how cell death occurs and spreads will help in the discovery of a treatment to reduce the impact of ischemia reperfusion damage.

In this study, we investigated the effect of Cx mimetic peptide Gap27 on Cx43 GJ protein, CxHC protein levels and GJIC in 3T3 fibroblasts under normal conditions. GAP27 aligns 100% with a part of the extracellular loop 2 of the Cx43 protein (Chaytor et al., 1997) and has consistently been identified as an effective inhibitor of GJIC. We show for the first time that this mimetic peptide can cause a rapid reduction in both Cx43 and CxHC protein levels in 3T3 fibroblasts. This leads to attenuation in GJIC as well as CxHC activity. Subsequently, we demonstrated in an *in vitro* model of ischemia reperfusion, that targeting Cx hemichannels can protect cells and minimise cell death.

## RESULTS

### Gap27 reduced Cx43 and CxHC levels in normal 3T3 fibroblasts

**Fig. 1.**
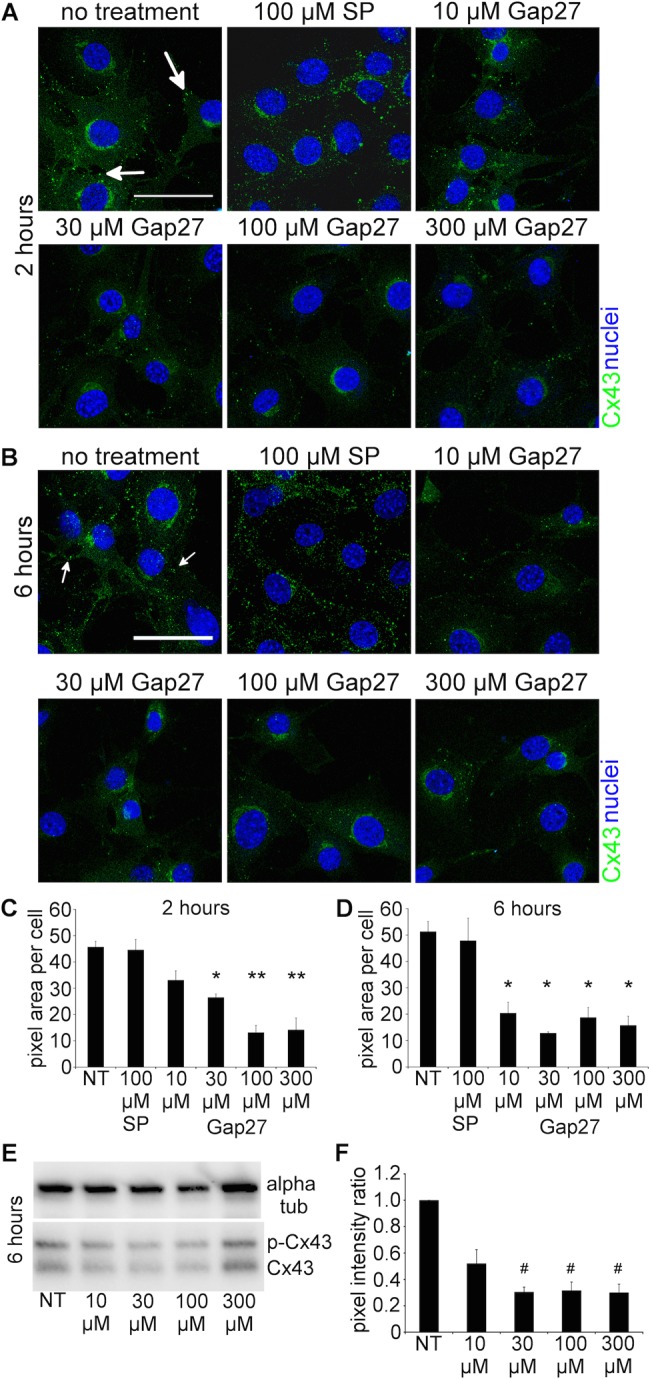
**Gap27 reduced Cx43 protein levels in normal 3T3 fibroblasts.** (A,B) Representative images of cells with no treatment (NT), 100 µM scrambled peptide (SP) treated, or treated with increasing doses of Gap27 for (A) 2 h or (B) 6 h. Cells were immunolabeled for Cx43 (green, white arrows) and nuclei (blue) identified by Höechst. (C,D) Cx43 labelling intensity was quantified at (C) 2 h and (D) 6 h. (E) Western blots of Cx43 protein levels at 6 h after Gap27 treatment. Alpha tubulin was included as a loading control housekeeping protein. (F) Intensity of the Cx43 band was presented in ratio to alpha tubulin. Data are means±s.e.m. (*n*=3). *P<0.01, **P<0.001, ^#^*P*<0.05 compared with NT, one-way ANOVA. Scale bar=50 µm.

To determine the effect of Cx mimetic peptide on Cx43 GJ and CxHC protein levels, 3T3 cells were treated with different doses of Gap27 for 2 h or 6 h. In cells with no-treatment or scrambled peptide (SP), Cx43 puncta were localised to the plasma membrane between neighbouring cells and the perinuclear region which corresponds to the Golgi compartment (Fig. 1A,B). After 2 h of incubation with Gap27 (30, 100, 300 μM), there was a significant reduction of Cx43 staining levels in comparison to no-treatment or scrambled peptide (Fig. 1A). A slight decrease was also seen in the cells treated with 10 μM of Gap27, although not statistically significant (Fig. 1C). In the cells treated with Gap27 for 6 h, Cx43 levels were significantly less for all the doses and reached about 60% reduction of Cx43 protein levels compared to no-treatment or scrambled peptide (Fig. 1D). This result was confirmed by western blotting (Fig. 1E,F). Since there was no difference between no-treatment and scrambled peptide, only no-treatment was included as a control in subsequent experiments.

**Fig. 2.**
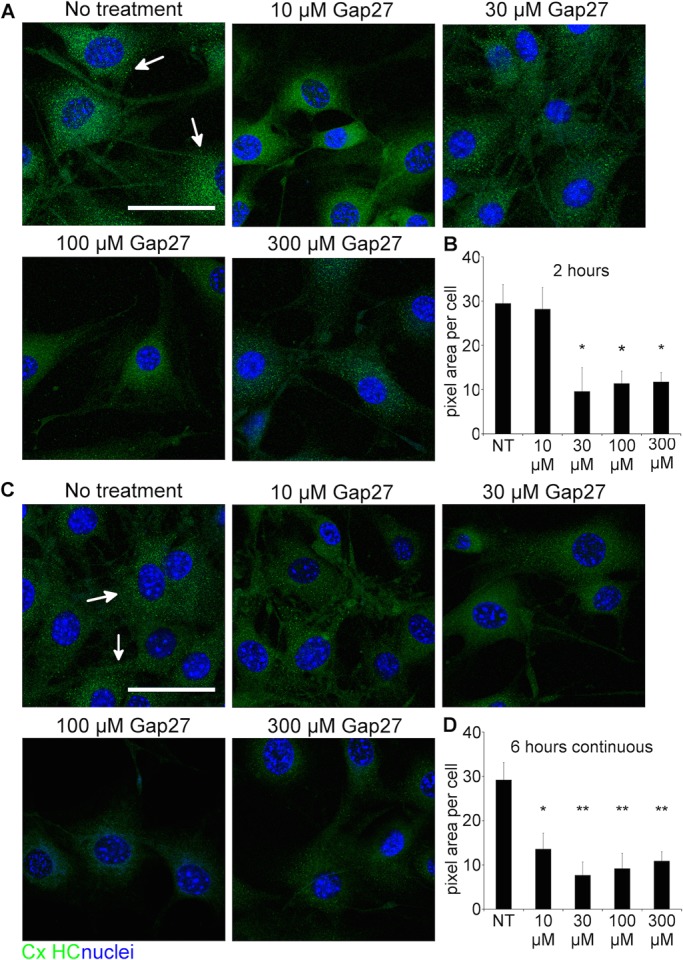
**Gap27 reduced CxHC protein levels in normal 3T3 fibroblasts.** (A,C) Representative images of 3T3 cells with no treatment (NT), or treated with increasing doses of Gap27 for (A) 2 h or (C) 6 h. Cells were immunolabelled for CxHC (green, white arrows) and nuclei (blue) identified by Höechst. (B,D) CxHC labelling intensity was quantified at (B) 2 h and (D) 6 h. Data are means±s.e.m. (*n*=3). *P<0.05, **P<0.01 compared with NT, one-way ANOVA. Scale bar=50 µm.

CxHC distribution patterns were studied using an antibody that only binds to undocked Cx. CxHC puncta were much smaller in size compared to the Cx43 labelling (Fig. 2). Similar to the observation with Cx43, less CxHC staining was detected in the cells treated with Gap27 compared to no-treatment at 2 h (Fig. 2A) and 6 h (Fig. 2C). Significant reduction in CxHC levels was seen with Gap27 (30, 100, 300 μM) after 2 h (Fig. 2B) and in all the doses after 6 h incubation (Fig. 2D).

### Gap27 significantly reduced GJIC between fibroblast cells

**Fig. 3.**
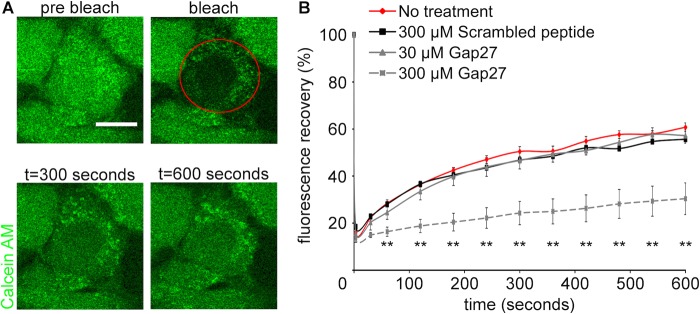
**High concentration of Gap27 reduced GJIC in normal 3T3 fibroblasts.** (A) Representative images of FRAP experiment in cells treated with Gap27 (30, 300 µM), scrambled peptide (300 µM), or no-treatment for 2 h. The bleached cell was circled in red. (B) The recovery profiles of fluorescence after FRAP. Data are means±s.e.m. (*n*=3). **P<0.01 compared with NT, one-way ANOVA. Scale bar=10 µm.

Fluorescence recovery after photobleaching (FRAP) was performed to assess the effect of Gap27 on GJIC between neighbouring cells (Fig. 3). Cells were pre-treated with Gap27 (30 or 300 μM) for 2 h prior to the FRAP experiment. Ten minutes after photobleaching of calcein, fluorescence recovery was ∼60% in cells with no-treatment or scrambled peptide (300 μM), whereas Gap27 (300 μM) treated cells showed ∼30% fluorescence recovery. This suggests that GJIC has been attenuated by Gap27 (300 μM). Such attenuation was not observed in cells treated with Gap27 (30 μM).

### The *in vitro* model of ischemia reperfusion with significantly reduced oxygen in culture media

To investigate the GJIC and CxHCs activity during ischemia reperfusion stress, a well characterised *in vitro* model of ischemia reperfusion was replicated (Pringle et al., 1997). Fibroblast cells were subjected to oxygen-glucose deprivation followed by reperfusion in glucose and reoxygenation (OGDR). Partial pressure of oxygen within the OGDR media was measured using the fibre oxygen (FOXY) probe, and the oxygen levels were reduced from 20% to less than 5% (Fig. S1).

### Cx43 and CxHC protein levels significantly increased in fibroblast cells after OGDR insult

**Fig. 4.**
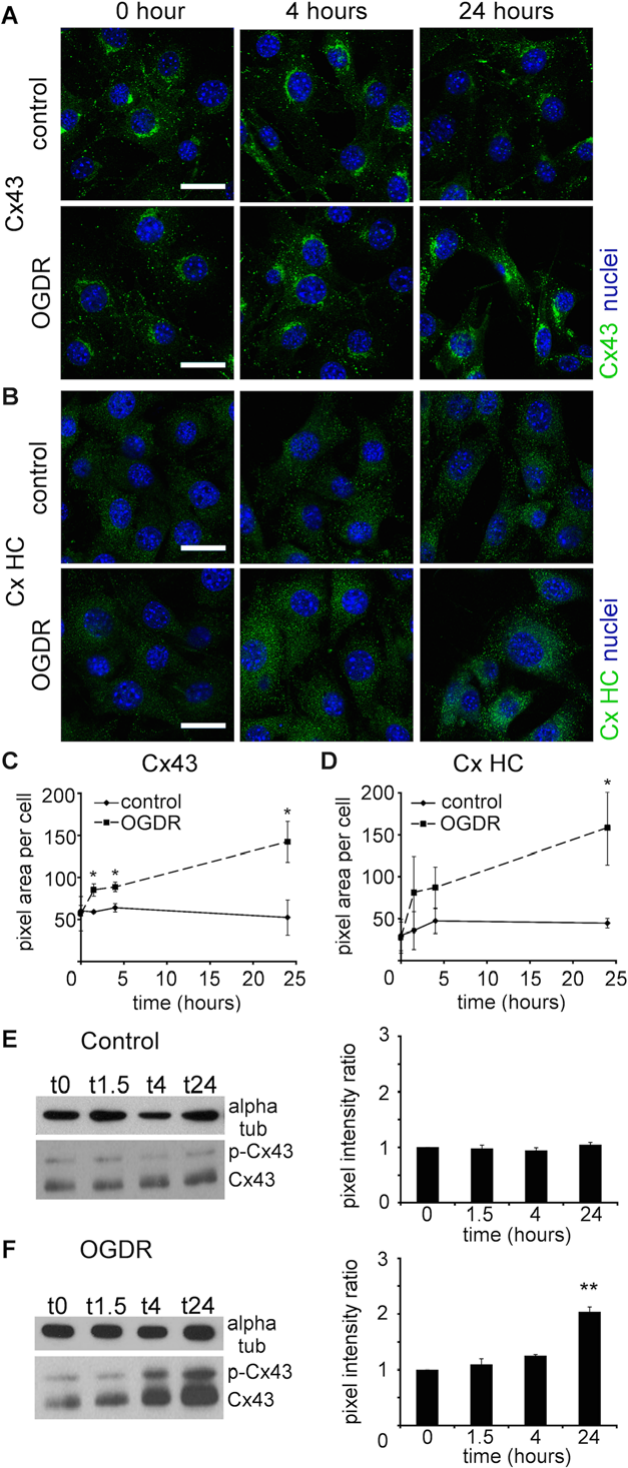
**OGDR insult significantly increased Cx43 and CxHC protein levels in 3T3 fibroblasts.** (A,B) Representative images of (A) Cx43 (green) and (B) CxHC (green) proteins in control 3T3 fibroblasts and that have undergone OGDR stress. (C,D) Labelling intensity of (C) Cx43 and (D) CxHC were quantified. Data are means±s.e.m. (*n*=4). *P<0.05, *t*-test. (E,F) Western blots and quantification of (E) Cx43 in control cells and (F) cells subjected to OGDR insult (OGDR WT 3T3) and reperfused for various time period. Alpha tubulin was stained as loading control and used for normalisation. Pixel intensity of the bands was measured. Data are means±s.e.m. *n*=3, **P<0.01 compared with 0 h, one-way ANOVA. Scale bar=20 µm.

Cx43 and CxHC protein levels were examined in fibroblast cells under normal culture conditions and subjected to OGDR insult. In cells subjected to OGDR insult, increasing levels of Cx43 and CxHC were observed at 4 and 24 h after reperfusion and compared to the control cells (Fig. 4A-D). This was also confirmed by western blotting against Cx43 proteins (Fig. 4E-F).

### Gap27 prevented the increase of GJIC after OGDR insult

**Fig. 5.**
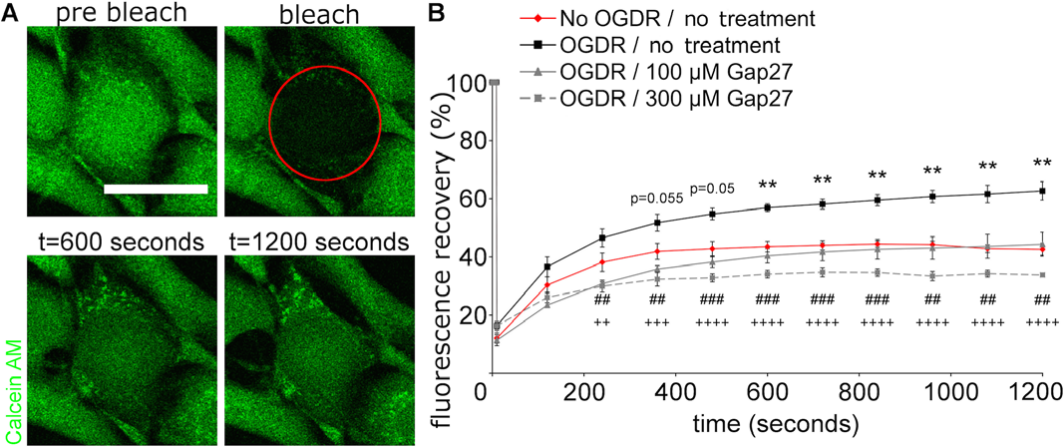
**High concentration of Gap27 prevented the perturbed GJIC at 4 h after OGDR insult.** (A) Representative images of the FRAP experiment. The bleached cell was circled in red. (B) The recovery profiles of fluorescence after FRAP. Data are means±s.e.m. (*n*=4). **P<0.01 compared with No OGDR, ^##^*P*<0.01, ^###^*P*<0.001, ^++^*P*<0.01, ^+++^*P*<0.001, ^++++^*P*<0.0001 compare with OGDR, one-way ANOVA. Scale bar=25 µm.

Since upregulation of Cx43 and CxHC levels were seen after OGDR insult, FRAP was performed to investigate GJIC between adjacent cells. In cells subjected to OGDR insult and reperfused for 4 h (Fig. 5, black line), the recovery rate of fluorescence after photobleaching was significantly faster than the normal fibroblasts (Fig. 5, red line), indicating increased GJIC. Treatment of Gap27 (100 μM; Fig. 5, grey solid line) after OGDR reverted the OGDR-induced increase whilst Gap27 (300 μM; Fig. 5, grey dash line) reduced the fluorescence recovery rate further than that seen in normal fibroblasts.

### Gap27 reduced the opening of CxHC after OGDR insult

**Fig. 6.**
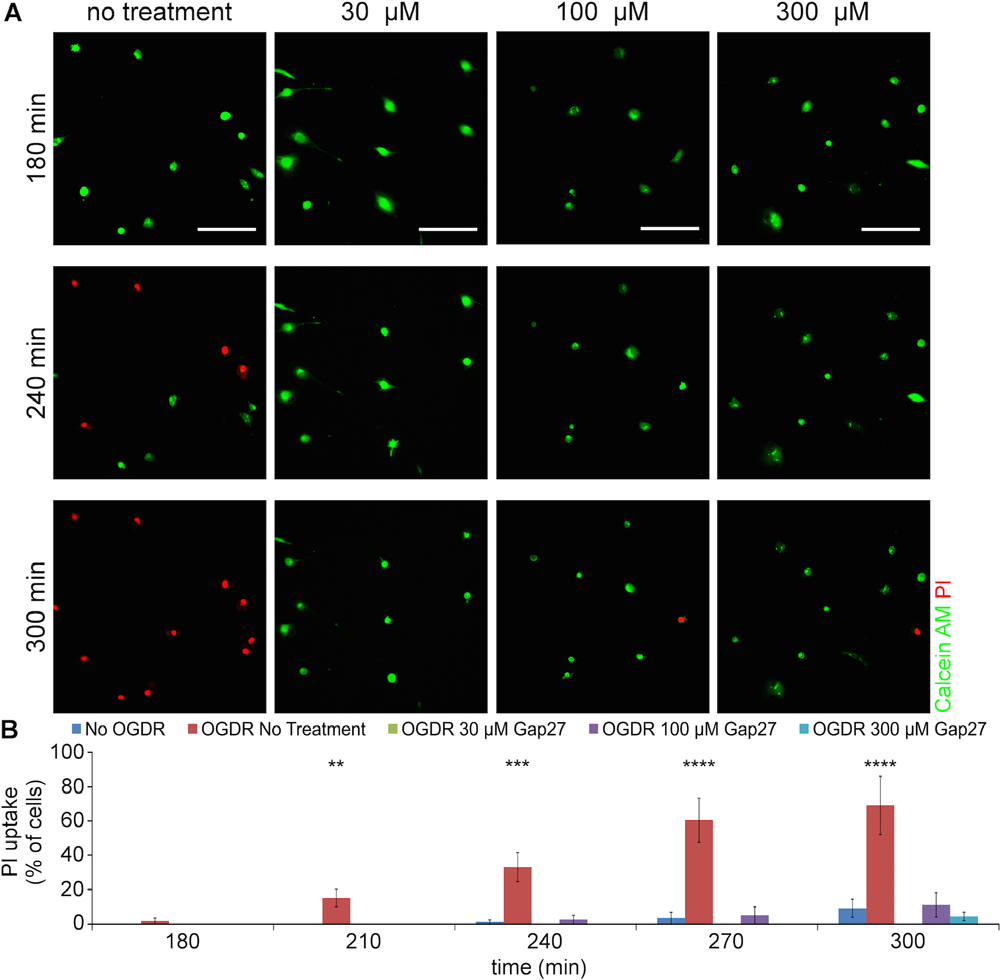
**PI uptake in low confluence cells subjected to OGDR insult and Gap27 incubation.** (A) Representative images of 3T3 fibroblasts after OGDR insult and Gap27 treatment, up to 5 h after OGDR. Live cells were identified by Calcein AM (green) and cells with open CxHC showed permeation of PI (red). (B) Quantification of PI-positive cells, as a percentage of Calcein-AM positive cells in the first frame of time-lapse. Data are means±s.e.m. (*n*=4). **P<0.01, ***P<0.001, ****P<0.0001 compare with No OGDR, one-way ANOVA. Scale bar=100 µm.

PI uptake assay was performed on low confluence fibroblasts to assess CxHC activity after OGDR insult. Cells were seeded at low confluency to minimise cell-to-cell contact and therefore CxHC at the plasma membrane remained undocked. PI is a positively charged dye which is normally excluded from live cells unless they open HC (or the cell membrane is compromised) and was used here as extracellular fluorescent permeability tracer to examine CxHC activity. Under normal culture conditions, almost all of the cells took up calcein-AM and converted it to calcein (Fig. 6B, No OGDR). OGDR stress caused more PI entry into the cells, peaking at 5 h after OGDR insult (Fig. 6, 300 min). At the same time, calcein signal disappeared. Incubation with Gap27 (all doses) significantly reduced the cellular uptake of PI with many cells remained calcein positive (Fig. 6), although some PI positive cells were still evident.

### Reducing Cx43 protein levels minimised cell death after OGDR insult

**Fig. 7.**
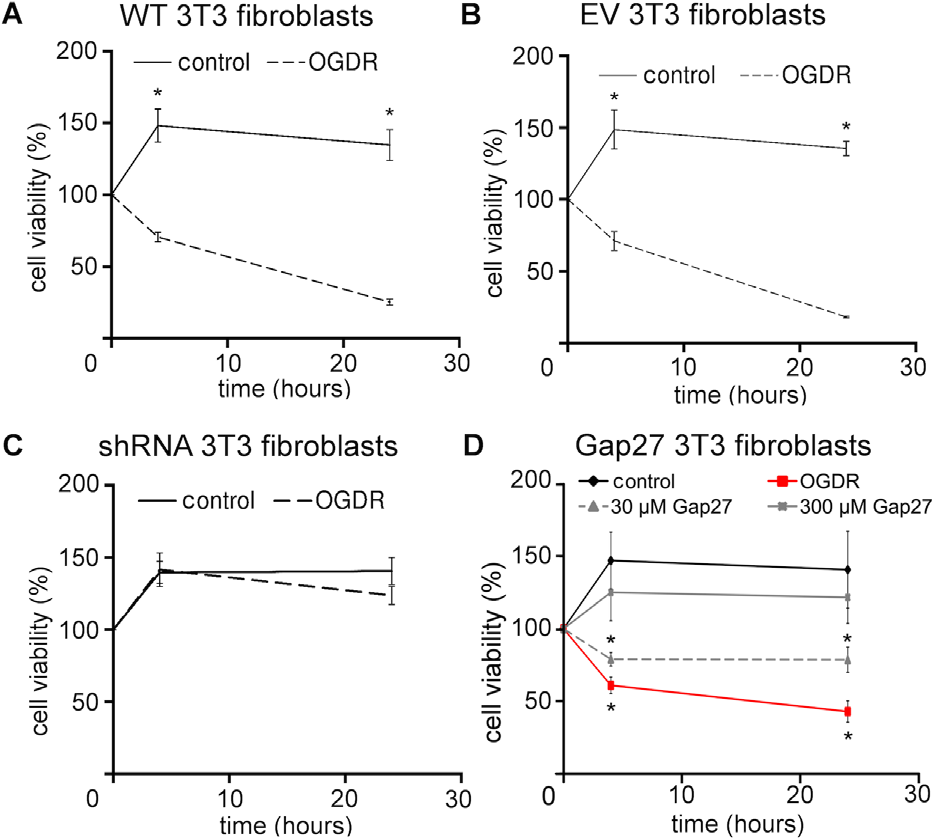
**Reducing Cx43 expression preserved cell viability at 4 h and 24 h after OGDR insult.** (A-C) Cell viability measurements of (A) wild-type 3T3 fibroblasts, (B) 3T3 fibroblasts transduced with empty vector (EV) or (C) Cx43shRNA. *n*=3, *P<0.05, *t*-test. (D) Cell viability measurements of wild-type 3T3 fibroblasts treated with Gap27 (30 μM or 300 μM). *n*=4, *P<0.05 compare with control, one-way ANOVA. Data are means±s.e.m.

Effect of the OGDR insult on cell viability was investigated in wild-type 3T3 fibroblasts or 3T3 fibroblasts with reduced Cx43 and CxHC protein levels following either Cx43shRNA transduction (Fig. S1) or Gap27 treatment. After OGDR insult, viability of wild-type fibroblasts significantly dropped at 4 h and further reduced at 24 h (Fig. 7A). Similar observation was noted for fibroblasts transduced with empty vector P.Sup (Fig. 7B). However, when Cx43 expression was reduced by either Cx43shRNA (Fig. 7C) or Gap27 (300 μM) treatment (Fig. 7D), cell viability was significantly increased to levels almost comparable to the cells without OGDR insult at both time points. Gap27 (30 μM) also showed higher cell viability in comparison to OGDR with no-treatment, but there was still considerable decrease in cell viability.

## DISCUSSION

In this study, we investigated the mechanism of action of a connexin extracellular loop mimetic peptide on GJ and CxHCs in normal fibroblasts *in vitro*. We showed, for the first time, that the representative mimetic class peptide, Gap27, can significantly reduce the levels of both CxHC and Cx43 GJ protein in a concentration dependent manner, and subsequently reduced GJIC. Although it has been shown by an electrophysiolgical approach that mimetic peptides bind to CxHC prior to blockage of GJ (Evans et al., 2012; Kwak and Jongsma, 1999), we provided evidence that Gap27 can downregulate CxHC protein levels (Fig. 2) and reduce CxHC activity (Fig. 6). Decreased CxHC expression can also, to some extent, account for the downregulation of Cx43 GJ protein as one precedes the other. Maximum reduction of CxHC levels was rapidly achieved at 2 h after mimetic peptide (30 μM) treatment, with 67% reduction for CxHC levels (Fig. 2B). At this time there was only a 40% reduction in Cx43 protein (Fig. 1C). Higher concentration (100-300 μM) of Gap27 (Fig. 1C) or longer incubation time (Fig. 1D) was required before further downregulation of Cx43 GJ could be achieved. In support of the immunocytochemistry result, reduced GJIC at 2 h post treatment was only seen with high concentration of mimetic peptide (300 μM) and not with the low concentration (30 μM) (Fig. 3).

In GJ formation, the two extracellular loops of Cxs from opposing cells interact and dock to form a channel. Each loop has three conserved cysteine residues joined by at least one disulphide bond. It is doubtful that mimetic peptides can disrupt GJ by breaking this strong bond. Indeed, it has been shown in cells transfected with fluorophore-tagged Cx that GJ internalise as a channel into just one of the two cells (Jordan et al., 2001). Electrophysiological studies using the mimetic peptide Gap26 also demonstrated that the CxHC is the first target of mimetic peptides, before further incubation prevents these CxHCs from docking to their neighbours and ultimately inhibiting GJIC (Desplantez et al., 2012).

The effects of the Gap27 mimetic peptide on Cx43 GJ protein levels have been reported in both *in vitro* and in *ex vivo* spinal cord segment models (Chen et al., 2015a; O'Carroll et al., 2008). A markedly reduced expression of Cx43 was observed when compared to the scrambled peptide. In HeLa cells stably transfected with Cx43-GFP, decreased GJIC was reported but without significant difference in the number of Cx43 plaques between cells treated with mimetic peptide and untreated control cells (Berman et al., 2002). In that study, however, the cells were treated with sodium butyrate for 18 h prior to the experiment to enhance protein expression and therefore cannot directly be compared to natural Cx43 expression in cells. In addition, in those studies the cells were only treated with mimetic peptide for 1.5 h, which may be too early to detect any obvious decrease in Cx43 expression given that the Cx43 half-life is about 2-3 h (Laird, 2006). Equally in some other studies, incubation with mimetic peptides for 1.5 h caused decreased GJIC but not Cx43 expression, which again might not be expected in this short time frame (Wright et al., 2009). The same group also reported similar finding in the rat aortic A7r5 smooth cell line following 4 h incubation with Gap26 or Gap27 treatments, although a downward trend was observed for the relative fluorescence levels of Cx43 in comparison to the controls (Martin et al., 2005). As other studies have performed similar experiments without using reduced serum media [it has been well documented that proteases within serum can rapidly digest the peptides (Wright et al., 2009)], the sera can greatly reduce the efficacy and longevity of the peptides. It is likely that the duration of treatment, experimental setup, and the cell types used will all play a role in the effect of mimetic peptide on Cx protein levels.

After establishing that the Gap27 mimetic peptide reduced CxHC levels and subsequently reduces Cx43 GJ levels and GJIC in our experiments, we investigated the therapeutic potential of mimetic peptide in an *in vitro* model of ischemia+reperfusion. A significant elevation of CxHC and Cx43 GJ protein levels was evident 24 h after OGDR insult (Fig. 4), concomitant with increased CxHC activity (Fig. 6) and GJIC (Fig. 5). These changes correlated with a decrease in cell viability (Fig. 7A), which we confirmed was largely a result of increased connexin-based communication. In cells transduced with Cx43shRNA, cell viability after OGDR was almost the same as without OGDR (Fig. 7C).

Our results are in agreement with other studies that have demonstrated reduced cell death after OGDR upon treatment with carbenoxalone (a non-specific inhibitor of gap junction communication) or Cx43-specific antisense (Frantseva et al., 2002). Based on studies in the heart and brain, it was proposed that metabolic inhibition from an ischemic episode opens CxHCs upon reperfusion, which accelerates cell death (Clarke et al., 2009; Contreras et al., 2002; Davidson et al., 2013; Johansen et al., 2011; Orellana et al., 2010; Thompson et al., 2006). In addition, increases in Cx43 expression and GJIC were suggested to play a role in the spread of damage through the ‘bystander’ effect when cell death signals spread laterally through GJ from dying cells to their healthy neighbours (Cotrina et al., 1998; Danesh-Meyer et al., 2012; Davidson et al., 2012; Mao et al., 2009; Zhang et al., 2013).

Similar to the various agents that minimise connexin expression and communication, the mimetic peptide rescued the cells after OGDR insult in a concentration dependent manner (Fig. 7D). CxHCs opened after OGDR and allowed PI to enter the cells over the period of reperfusion (Fig. 6). Although loss of membrane integrity may also lead to PI permeation, it is unlikely to be the case here because cells still appeared healthy with no signs of rounded morphology or detachment at 5 h after OGDR. The fact that PI permeation was prevented with treatment of both low and high concentration of Gap27 also indicates that PI enters into the cell through open CxHC. Cell viability had actually dropped by 4 h after OGDR (Fig. 7D) as shown by MTT assay. High concentration (100-300 μM) of Gap27 dampened GJIC (Fig. 5) thereby reducing cell death to some extent (Fig. 7D).

In conclusion, we have confirmed that Cx43 plays an important role in cell death during ischemia-reperfusion in fibroblast cells. This is in comparable to that described in experiments with heart and brain cells. We established that the Gap27 mimetic peptide could rapidly reduce CxHC protein levels within 2 h with concentration as low as 30 μM, while higher concentrations of at least 100 μM or longer treatment time is required for downregulation of Cx43 GJ protein. Reduction in CxHC and Cx43 GJ protein leads to a decrease GJIC and can protect the cells from ischemia reperfusion damage. The Gap27 mimetic peptide may serve as a potential therapeutic agent for treating connexin-based disorders and perhaps preventing the progression of pressure ulcer development by dampening the damage induce by repetitive episodes of ischemia reperfusion.

## MATERIALS AND METHODS

### Reagents

All chemicals and reagents were purchased from Sigma-Aldrich unless otherwise stated. Mimetic peptide Gap27 shares a conserved sequence homology to the second extracellular loop (E2) of Cx (amino acid 204-214; SRPTEKTIFII) and was custom made with 98% purity (Thermo Scientific). Scrambled peptide (SP) (RFKSPSLCTTDEV) was based on a previous publication (O'Carroll et al., 2008). Peptides were prepared in reduced serum media Opti-Minimal Essential Medium (OptiMEM) supplemented with 1% donor bovine serum (DBS), abbreviated to OptiMEM+. OptiMEM+ allowed cells to continue to grow and proliferate while minimising breakdown of mimetic peptide by proteases.

Rabbit anti-CxHC is a custom made antibody (affinity purified) against a highly conserved region of the first extracellular loop (E1) of Cx, sequence ESAWGDEQSAFRCNTQQPGC.

Rabbit anti-Cx43 was purchased from Sigma-Aldrich (C6219).

### Cell culture, retroviral constructs, and transduction

Mouse fibroblast cells, NIH 3T3 (ATCC #CRL-1658) were cultured with Dulbecco's Modified Essential Medium (DMEM) supplemented with 10% DBS, 100 U/ml penicillin and 100 µg/ml streptomycin in 5% CO_2_ and 95% air at 37°C. 3T3 cells were transduced with aCx43-specific shRNA (Cx43shRNA; sequence GGTGTGGCTGTCAGTGCTC) construct gifted by W. H. Moolenaar (van Zeijl et al., 2007), or a retroviral empty vector pSuppressor (p.Sup) from Imgenex. The GP2-293 packaging cell line (Clonetech) was transfected by calcium phosphate precipitation with 5 µg pMD.G envelope plasmid, 10 µg pBSII SK-carrier plasmid (Stratagene), and 15 µg retroviral plasmid containing either Cx43shRNA or the empty vector p.Sup. Viral media was collected to infect the 3T3 cells for 48 h, with fresh viral media being replaced every 12 h. Transduced cells were selected based on resistance to 2 µg/ml puromycin (Cx43shRNA) or 500 µg/ml geneticin (empty vector). The first three passages of transduced cells were used for experiments.

### Mimetic peptide incubation

When cells were subjected to 2 h incubation, a single dose of Gap27 (10, 30, 100, 300 µM) or scrambled peptide (100 µM) was applied. For 6 h incubation, fresh media containing peptides was replaced every two hours. A control without peptide was included.

### *In vitro* ischemia reperfusion model

Cells were subjected to ischemia reperfusion by exposing them to oxygen-glucose deprivation/reoxygenation (OGDR). Briefly, DMEM (glucose and serum free) that had been effervesced with 5% CO_2_ and 95% N_2_ for 1 h was applied to the cells. Cells were then placed inside a sealed chamber with an atmosphere of 5% CO_2_ and 95% N_2_ for 1.5 h, followed by re-oxygenation in fresh OptiMEM+ at 37°C in 5% CO_2_ and 95% air for 0, 1.5, 4 or 24 h. Oxygen levels in the media were measured using a fibre optic oxygen (FOXY) probe (Ocean Optics).

### Immunofluorescence

Cells were grown to confluence in 8-well chamber slides (Millicell EZ slide, Millipore) and subjected to treatments. Cells were then fixed in 4% paraformaldehyde for 15 min, permeablised with 0.1% Triton X-100 for 15 min, and blocked with 0.1 M lysine-PBS for 30 min. Rabbit anti-Cx43 (1:4000) was applied for 1 h at room temperature or rabbit anti-CxHC (1:1000) overnight at 4°C, followed by goat anti-rabbit Alexa 488 (1:500; Life Tech) for 1 h. A mixture of Höechst 33258 and 33342 (1:50,000) was applied to stain the nuclei. Slides were coverslipped in Citifluor and imaged under a confocal microscope (Upright SPE, Leica). All images were acquired using identical parameters. Cx or GJ profile was analysed using ImageJ (version 1.46r, Wayne Rasband, NIH) by setting a threshold to binary segregate connexin puncta and background, and the number of nuclei was counted. All images were processed using identical parameters and data was presented as connexin pixel area per cell.

### Western blots

Cells were grown to confluence in 6-well plates and subjected to treatments. Cells were lysed using cold radio immunoprecipitation (RIPA) buffer supplemented with protease and phosphatase inhibitor cocktail (Roche). Lysates were sonicated for 30 min, centrifuged at 4°C for 10 min and the supernatant was collected. Total proteins were measured by BCA assay (Thermo Scientific). Equal amounts of protein were denatured with Laemmli buffer (Bio-Rad) and resolved in 10% SDS-PAGE gel (Bio-Rad), then transferred to nitrocellulose membrane. The membrane was blocked in 1% non-fat milk in PBS-Tween for 1 h at room temperature, and incubated overnight at 4°C with rabbit anti-Cx43 (1:4000). Subsequently, goat anti-rabbit (1:1000) conjugated with horseradish peroxidise was applied for 1 h. Alpha tubulin (1:2000 for 1 h; Abcam, ab64332) was used as a housekeeping protein. Signal was detected using West Pico Chemilluminescent Substrate (Thermo Scientific) and imaged on a ChemiDoc™ (Bio-Rad). Band intensity was analysed using ImageJ. All protein collection for western blots was repeated in triplicate.

### Cell viability assay – MTT

10,000 cells per well were cultured in 96-well plates and subjected to OGDR insult, followed by the MTT assay [3-(4, 5-dimethylthiazol-2-yl)-2, 5-diphenyl tetrazolium bromide] according to the manufacturer's instructions (Millipore). Briefly, 10 µl of MTT solution (5 mg/ml in PBS) was added to the culture media in each well. After 4 h of incubation at 37°C, 100 µl of isopropanol with 0.04 N hydrochloric acid (HCl) was added to each well. Absorbance was measured on a microplate spectrophotometer (Spectra Max 340; Molecular Devices) at a test wavelength of 570 nm and a reference wavelength of 630 nm. Absorbances were normalised to the untreated cells at 0 h after reperfusion (i.e. 100% viability) and presented as percentage of viability.

### Communication assay – fluorescent recovery after photobleaching (FRAP)

FRAP was performed to investigate GJIC between adjacent cells. Cells were grown to confluence in glass bottom 35 mm^2^ dishes and subjected to OGDR insult. After the insult, cells were incubated with Gap27 (100 or 300 µM) or left untreated in optiMEM+. A control without OGDR was included. After 4 h of treatment, calcein-AM (1 µl/1000 µl media) was applied for 20 min. The cells were then washed 3× in PBS and replaced with fresh OptiMEM+ containing propidium iodide (PI) and Gap27 or OptiMEM+ only. FRAP images were taken on a confocal microscope with temperature and gas controlled chamber (5% CO_2_ and 95% air at 37°C). A zoomed region of interest (ROI) covering a connected cell was bleached of its fluorophore with high laser power and subsequently imaged at a rate of 1.635 s per frame. The speed of fluorescent recovery was quantified using ImageJ.

### CxHC activity assay during OGDR

PI uptake assay was used to assess CxHC activity. Cells were grown to low confluence to ensure no cell-to-cell contact and therefore CxHC on the plasma membrane remained undocked. Cells were subjected to OGDR insult, followed by 20 min incubation with calcein-AM. The cells were then washed 3× in PBS and replaced with fresh OptiMEM+ containing PI and Gap27 (30, 100 or 300 µM) or OptiMEM+ alone. A control with no OGDR insult was included. Images were taken on an Olympus inverted microscope (Olympus 1X81) with temperature and gas controlled chamber (5% CO_2_ and 95% air at 37°C) every 30 min for 5 h. PI positive cells were counted and expressed as a percentage of calcein positive cells in the first frame taken.

### Bystander effect assay during OGDR

Cells were grown to confluence to ensure GJ formation between adjacent cells. After OGDR insult, cells were treated with Gap27 (10, 30, 100, 300 or 1000 µM), 100 µM scrambled peptide, or left untreated in OptiMEM+ only. A control without OGDR insult was included. After 4 h of treatment, calcein-AM was applied for 20 min. The cells were then washed 3× using PBS and replaced with OptiMEM+ containing PI and Gap 27or OptiMEM+ only. Images were taken on the Olympus 1X81 microscope with temperature and gas controlled chamber (5% CO_2_ and 95% air at 37°C) every hour for 24 h. PI positive cells were counted and expressed as a percentage of calcein positive cells in the first frame taken.

### Statistics

Statistical comparisons were made using either a one-way analysis of variance (ANOVA) or independent-samples *t*-test using SPSS Statistics (version 21, IBM). Data was tested for normality (Shapiro–Wilk test) and equal variances (Levene test) before statistical comparisons were carried out. In time-lapse experiments, the null hypothesis was rejected using a multivariate analysis of variance (MANOVA). The Dunnett post hoc test (against the control sample) or Tukey post hoc test were performed if data proved significant. All experiments were independently repeated for 3×. All data are presented as mean±s.e.m.
